# Supplementary material for: Development of an Automated Online Flow Cytometry Method to Quantify Cell Density and Fingerprint Bacterial Communities
Source: Cells. 2023 Jun 6;12(12):1559. doi: 10.3390/cells12121559 (PMC10296507; doi:10.3390/cells12121559)
Supplement: Supplementary file 1 [file cells-12-01559-s001.zip › cells-2384374-supplementary.pdf]

Development of an automated online flow cytometry method to quantify cell density and fingerprint bacterial communities

Juan López-Gálvez[a], Konstanze Schiessl[b], Michael Besmer[b], Carmen Bruckmann[a], Hauke Harms[a] and Susann Müller[a*].

[a]Helmholtz-Centre for Environmental Research, Department of Environmental Microbiology, Permoserstraße 15, D-04318 Leipzig, Germany

[b] onCyt Microbiology AG, Marchwartstrasse 61, 8038 Zürich, Switzerland

*Corresponding author: Susann Müller, Helmholtz-Centre for Environmental Research, Department of Environmental Microbiology, Permoserstraße 15, D-04318 Leipzig, Germany
Email: susann.mueller@ufz.de

List of contents

SI 1: Preparation of reagents. 2

SI 1.1: LB medium. 2

SI 1.2: LB medium agar. 2

SI 1.3: PBS buffer. 2

SI 1.4: PFA fixation solution. 2

SI 1.5: DAPI staining solution. 2

SI 2: Supplementary Figures. 2

SI 2.1 Calibration of the flow cytometer CytoFLEX. 2

SI 2.2. Comparison of mock community (MC) fingerprints after standard PFA/EtOH fixation and DAPI staining and after the new NaCl/NaN_3/EtOH fixation and DAPI staining method. 3

SI 2.3. Comparison of DAPI patterns of live *K. rhizophila* after cleaning. 4

SI 2.4. Cell concentration over time of live cells of the MC. 4

SI 2.5. Cell gating using cyPlot software for cell concentration calculation of online analysed MC using pre-fixed cells. 5

SI 2.6. Manual gating of the MC for calculation of proportions of different subpopulations *of P. polymyxa*. 6

SI 2.7. Comparison between DAPI patterns of the MC after PFA/EtOH fixation and NaCl/NaN_3/EtOH fixation measured by the Influx. 6

SI 2.8. Comparison of repeated measurements of MC fingerprints. 7

SI 2.9. NMDS plot of repeated measurements of MC vs. pure strain fingerprints. 8

SI 2.10. Comparison of FSC vs SCC patterns of live *K. rhizophila*. 8

SI 3: Movies. 9

Movie SI 3.1 9

Movie SI 3.2 9

Movie SI 3.3 9

Movie SI 3.4 9
Datasets 10
References 11

Supplementary information.

SI 1: Preparation of reagents.

SI 1.1: LB medium.

Mix yeast extract 5 g/L, NaCl 5 g/L and tryptone 10 g/L with double distilled water and adjust to pH 7.0.

SI 1.2: LB medium agar.

Add agar to reach a concentration of 20 g/L to the LB medium.

SI 1.3: PBS buffer.

Mix 6 mM Na_2HPO_4, 1.8 mM NaH_2PO_4 and 145 mM NaCl in double distilled water, adjust to pH 7.0 and autoclave.

SI 1.4: PFA fixation solution.

Add 4 g of PFA (paraformaldehyde) in 50 mL of PBS buffer and heat to 70 °C. Adjust pH to 7 with 37 % HCl solution. This stock solution is stored at -4 °C for maximum of 6 months. Dilute this stock to 2 % PFA in PBS for the fixation of cells.

SI 1.5: DAPI staining solution.

Mix 1 µM DAPI and Na_2HPO_4/NaH_2PO_4 buffer (289 mM Na_2HPO_4 and 128 mM NaH_2PO_4 with double distilled water, pH 7).

SI 2: Supplementary Figures.

SI 2.1 Calibration of the flow cytometer CytoFLEX.

Before starting any experiment, gains for the forward scatter, side scatter and DAPI fluorescence channels are calibrated by using an 0.5 µm and 1 µm UV bead mix (Polysciences, Cat. No. are 18339 and 17458, Warrington, PA, United States). The lower signals represent the instrumental noise from which the cells must be separated.

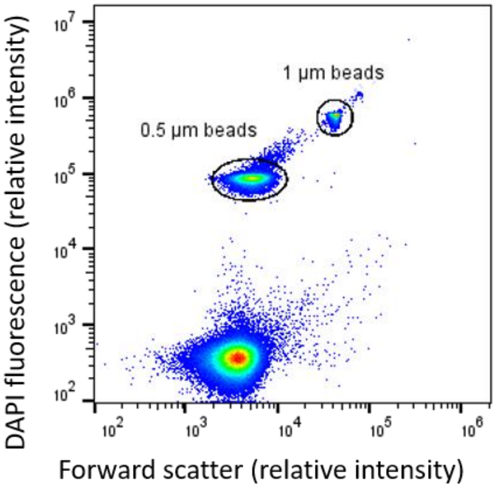

Figure SI 2.1. Calibration of the CytoFLEX. DAPI fluorescence against forward scatter of 0.5 µm and 1 µm UV Fluoresbrite Microspheres (Polysciences, Cat. No. are 18339 and 17458, Warrington, PA, United States) used for alignment of the CytoFLEX.

SI 2.2. Comparison of mock community (MC) fingerprints after standard PFA/EtOH fixation and DAPI staining and after the new NaCl/NaN$_3$/EtOH fixation and DAPI staining method.

PFA/EtOH fixation is the standard fixation procedure used by Cichocki et al. (2020), and NaCl/NaN$_3$/EtOH fixation is the new procedure that matches the capabilities of the OC-300 and the automated online workflow developed for the CytoFLEX. The PFA/EtOH fixation was followed by the standard DAPI staining procedure as described in Cichocki et al. (2020) while the new NaCl/NaN$_3$/EtOH fixation was followed by the new DAPI staining procedure (1 µM stock solution) for online analysis. In both cases, a clear separation of all three strains that make up the MC was achieved.

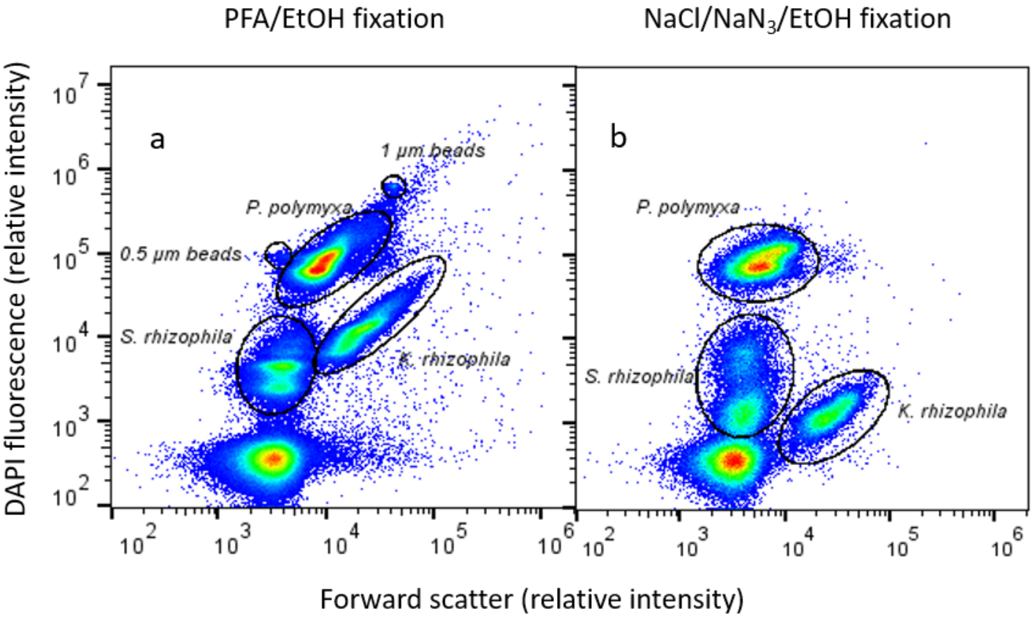

Figure SI 2.2. Analysis of the MC using 2 different fixation and staining methods. a) standard PFA/EtOH fixation and DAPI staining measured by the CytoFLEX. b) $NaCl/NaN_3/EtOH$ fixation and 1 µM DAPI stock solution measured online by the CytoFLEX.

SI 2.3. Comparison of DAPI patterns of live *K. rhizophila* after cleaning.

The cleaning of the OC-300 CytoFLEX instrumentation was tested with four different solutions (main text). Only the 1 % and the 10 % FlowClean cleaning agent solution were found to keep the carryover low. However, the use of the 100 % and 10 % FlowClean cleaning agent solution caused a change in the DAPI / FSC fingerprint of *K. rhizophila* while with the use of the 1 % cleaning solution the pattern remained unaltered. This is evidenced by the sample obtained by the manual fixation procedure, where no cleaning solution is used, as the sample is measured directly in the CytoFLEX without using the OC-300 (Figure 3e).

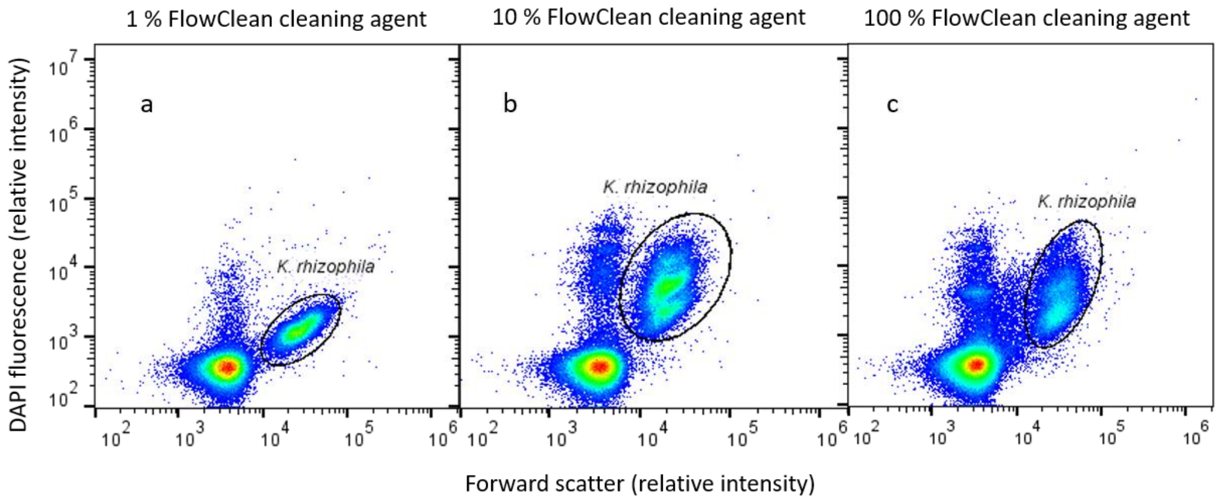

Figure SI 2.3. a) Pattern of live *K. rhizophila* when the automated measurement is performed using 1 % FlowClean cleaning agent. b) Pattern of *K. rhizophila* when the automated measurement is performed using 10 % FlowClean cleaning agent. c) Pattern of *K. rhizophila* when the automated measurement is performed using 100 % FlowClean cleaning agent.

SI 2.4. Cell concentration over time of live cells of the MC.

A sample of live cells of the MC was agitated in a flask filled with 50 mL PBS at RT. The OC-300 CytoFLEX instrumentation was used to perform the online automated flow cytometry workflow (main text, point 2.7) to measure the cell count for each of the contained three strains over 48 h. From the sample a proportion was taken and diluted, fixed, stained and measured every 45 min including the cleaning step to avoid carryover of previous measurements. The outcome shows that live cells of the MC, although kept in buffer instead of medium are not constant in their proportions and do change. In the main text we show that pre-fixed cells produces more stable data. However, it is clear from these data that live bacterial strains in PBS are active and change their proportions of subpopulations over time. This can also be observed in the Movie SI 3.1.

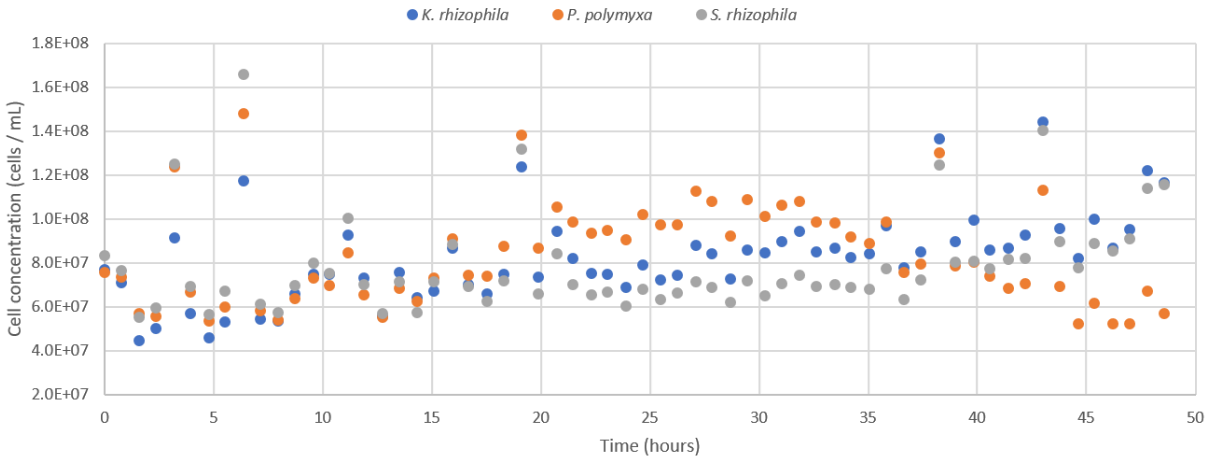

Figure SI 2.4. Live cell proportions for each strain of the MC were analyzed online using the OC-300 coupled to the CytoFLEX with the online automated workflow.

SI 2.5. Cell gating using cyPlot software for cell concentration calculation of online analysed MC using pre-fixed cells.

The raw data from the experiment shown in Figure 4 (main text) were gated for each strain of the MC using the cyPlot software to calculate the respective cell counts. The strains were pre-fixed by the standard procedure (Cichocki et al., 2020) before running the new online workflow by using NaCl/NaN_3/EtOH fixation and 1 µM DAPI stock solution (main text, point 2.7). The whole data set is shown in Movie SI 3.2. Two further parallel measurements are presented in Movie SI 3.3 and Movie SI 3.4.

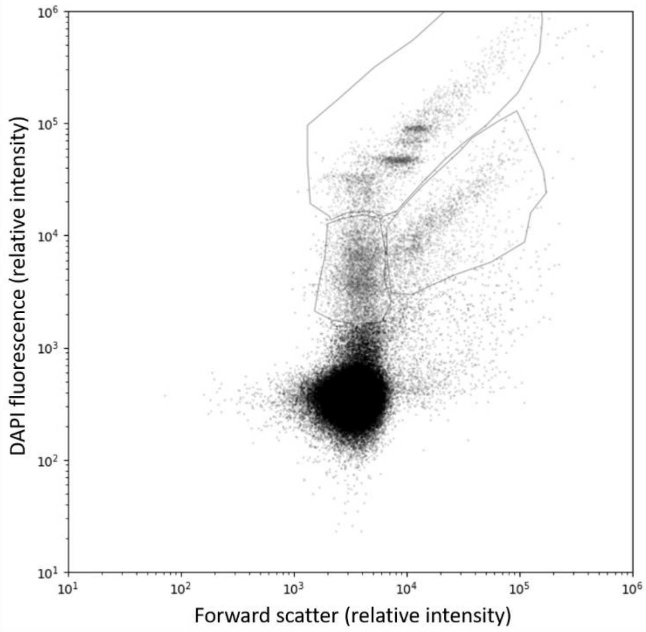

Figure SI 2.5. Cell gating done in the cyPlot analysis software used for the cell concentration calculation of the automated experiments in Figure 4 (main text). The sample originates from movie 3.2, 0h.

SI 2.6. Manual gating of the MC for calculation of proportions of different subpopulations *of P. polymyxa*.

On the left is shown the gating done using the software FlowJo for the discernment of the three strains of the MC. On the right, the gating was done only for the *P. polymyxa* strain to calculate the proportions of the C1, C2 and Cx subpopulations.

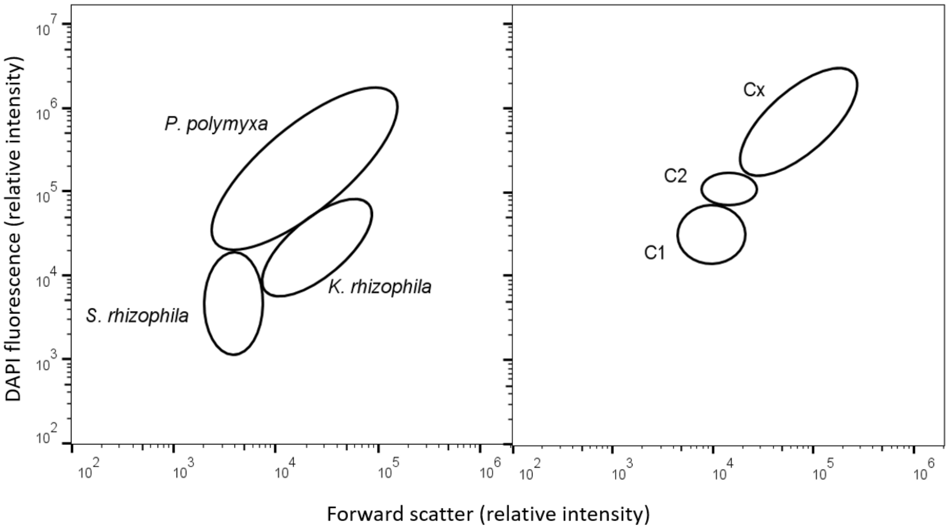

Figure SI 2.6. Gate template set in FlowJo software for the calculation of proportions of subpopulations of *P. polymyxa* shown in Fig. 6d. On the left, the gating for the three strains of the MC is shown. On the right, the gating for the three subpopulations within *P. polymyxa.*

SI 2.7. Comparison between DAPI patterns of the MC after PFA/EtOH fixation and NaCl/NaN_3/EtOH fixation measured by the Influx.

The PFA/EtOH fixation is the standard fixation and staining procedure used in Cichocki et al. (2020), which requires several centrifugation steps and overnight staining. The NaCl/NaN_3/EtOH fixation is the new procedure suited for the capabilities of the OC-300 CytoFLEX instrumentation and automated online flow cytometry. Both samples were measured using the Influx (BD Biosciences, Franklin Lakes, New Jersey, U.S.) to test if this high-end flow cytometer and cell sorter is also able to differentiate the 3 strains of the MC after applying the new method. Although the resolution of the MC was better with the standard method (SI 2.7a), the three strains of MC could also be distinguished with the method developed for the OC-300 CytoFLEX instrumentation (SI 2.7b).

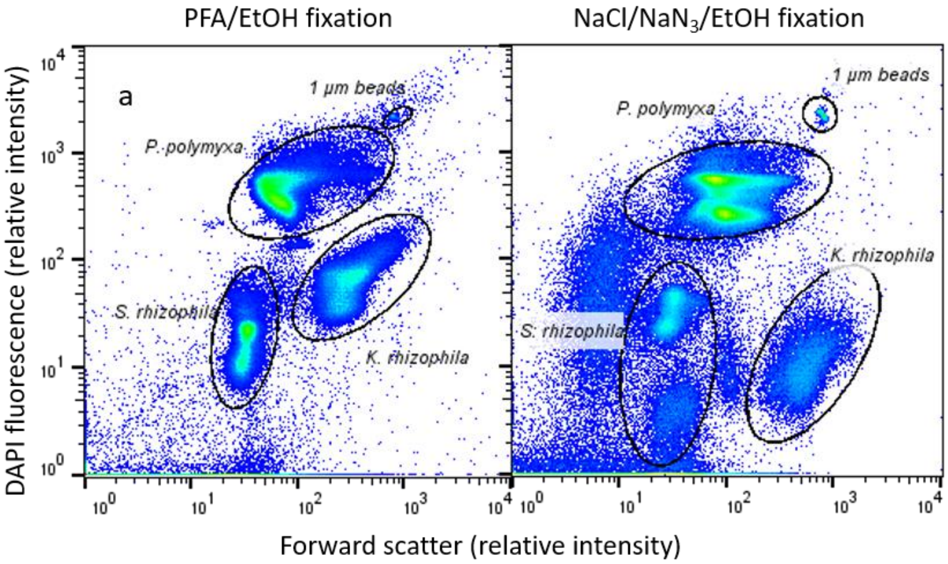

Figure SI 2.7. Fingerprints of the MC using 2 different fixation and DAPI staining methods. a) Fixation and staining according to the standard procedure of Cichocki et al. (2020), (b) Fixation with $NaCl/NaN_3/EtOH$ fixation and 1 µM DAPI stock solution, both measured using the Influx.

SI 2.8. Comparison of repeated measurements of MC fingerprints.

To determine the variability between measurements of the identical sample of live MC cells, the cells were stabilised in PBS, fixed with $NaCl/NaN_3/EtOH$ and stained with DAPI (1µM stock solution), manually.

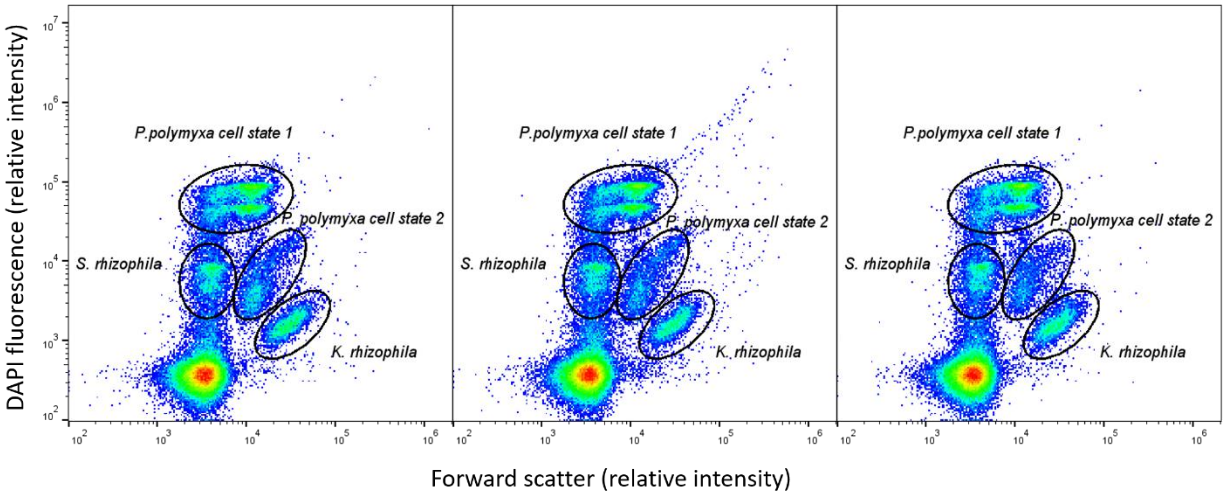

Figure SI 2.8. Triplicate of live cells of the MC using NaCl/NaN3/EtOH fixation and 1 µM DAPI staining measured by the CytoFLEX. The sample originated from Movie 3.1, 0h.

SI 2.9. NMDS plot of repeated measurements of MC vs. pure strain fingerprints.
A NMDS plot was created from the samples presented in section in section SI 2.8, as well as the individual strains that conform the MC. The NMDS plot was created using the flowCHIC tool [2] to show the divergence of the composition of the MC with respect to each other as well to the individual strains. The variation between the repeated measurement of the MC was found to be low but not identical.

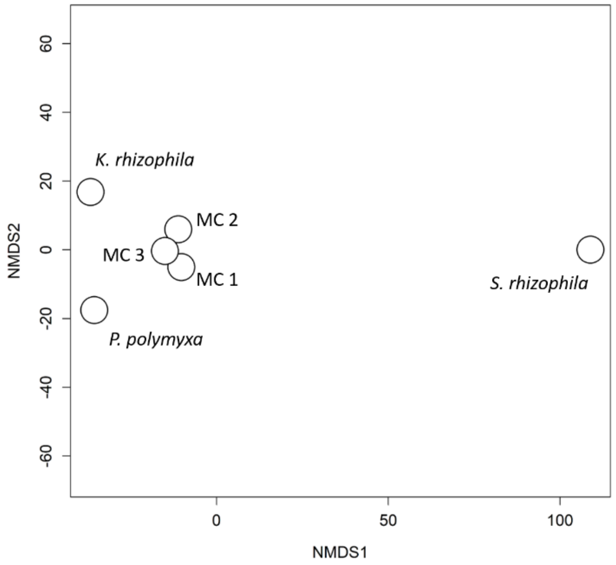

Figure SI 2.9. Divergence of the composition of the MCs, processed manually from live cells (section SI 2.8), as well as the individual sample of each strain visualized by a NMDS plot. The NMDS plot was created with the bioinformatics tool flowCHIC [2].

SI 2.10. Comparison of FSC vs SCC patterns of live *K. rhizophila*.
The cleaning of the OC-300 CytoFLEX instrumentation was tested with four different solutions (main text). Only the 1 % and the 10 % FlowClean cleaning solution were found to keep the carryover low. However, the use of the 100 % FlowClean cleaning agent solution caused a change in the SSC / FSC fingerprint of *K. rhizophila* while with the use of the 1 % cleaning solution the pattern remained unaltered compared to the pattern obtained by the new online fixation procedure (Figure 3).

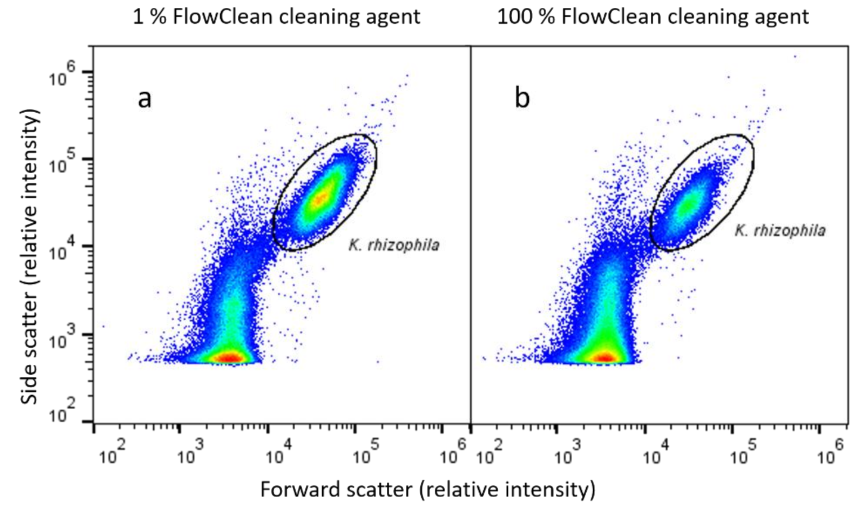

Figure SI 2.10. a) Pattern of live *K. rhizophila* when the automated measurement is performed using 1 % FlowClean cleaning agent. b) Pattern of *K. rhizophila* when the automated measurement is performed using 100 % FlowClean cleaning agent. The amount of background particles increased by 26.4 % when using the 100 % FlowClean solution.

SI 3: Movies.

Movie SI 3.1. Movie of samples obtained with a sample of live MC cells shaken in PBS for 48 h (Fig. SI 2.4). The OC-300 CytoFLEX instrumentation performed the online automated flow cytometry workflow to measure the cell counts for each of the contained three strains. From the sample a proportion was taken and diluted, fixed, stained and measured every 45 min including the cleaning step to avoid carryover of previous measurements.

Movie SI 3.2. Movie generated from patterns obtained from the first replicate of the automated online experiment using prefixed PFA/EtOH cells of the MC as sample over a period of 34 h (Fig. 4). The OC-300 CytoFLEX instrumentation performed the automated online flow cytometry workflow to measure the cell concentration for each of the contained three strains over 34 h. From the sample a proportion was taken and diluted, fixed, stained and measured every 45 min including the cleaning step to avoid carryover of previous measurements.

Movie SI 3.3. Movie generated from patterns obtained from the second replicate of the automated online experiment using prefixed PFA/EtOH cells of the MC as sample over a period of 34 h (Fig. 4). The OC-300 CytoFLEX instrumentation performed the automated online flow cytometry workflow to measure the cell concentration for each of the contained three strains over 34 h. From the sample a proportion was taken and diluted, fixed, stained and measured every 45 min including the cleaning step to avoid carryover of previous measurements.

Movie SI 3.4. Movie generated from patterns obtained from the third replicate of automated online experiment using prefixed PFA/EtOH cells of the MC as sample over a period of 34 h (Fig. 4). The OC-300 CytoFLEX instrumentation performed the automated online flow cytometry workflow to measure the cell concentration for each of the contained three strains over 34 h. From the sample a proportion

was taken and diluted, fixed, stained and measured every 45 min including the cleaning step to avoid carryover of previous measurements.

Datasets

Figure 3. Comparison of the patterns of three pure strains, harvested in the stationary phase of growth, when performed automatically (*K. rhizophila*, *P. polymyxa*, *S. rhizophila)*: https://dataverse.harvard.edu/privateurl.xhtml?token=a36e5b72-18cc-4d60-a25b-72ad63f8d360

Table 1. Testing of cleaning performance of four different cleaning solutions: MilliQ H_2O, undiluted 100 % FlowClean cleaning agent, 10 % FlowClean cleaning agent and 1 % FlowClean cleaning agent using three different strains: *P. polymyxa*, *K. rhizophila*, and *S. rhizophila*.

K. rhizophila, MilliQ H_2O:https://dataverse.harvard.edu/privateurl.xhtml?token=d69c549c-af6a-4bc6-b821-07a2b1035d3d

K. rhizophila, 100% FlowClean cleaning agent: https://dataverse.harvard.edu/privateurl.xhtml?token=8ab17d92-bd43-4aae-abfe-ce3481a35063

K. rhizophila, 10% FlowClean cleaning agent: https://dataverse.harvard.edu/privateurl.xhtml?token=01164aa5-7d5e-4563-b967-0f962f5e42c5

K. rhizophila, 1% FlowClean cleaning agent: https://dataverse.harvard.edu/privateurl.xhtml?token=0d1e064b-8bb9-4452-b676-2e63f8c3d29f

S. rhizophila, MilliQ H_2O: https://dataverse.harvard.edu/privateurl.xhtml?token=88e30329-7477-4080-b41d-500a2cdba1ee
S. rhizophila, 100% FlowClean cleaning agent: https://dataverse.harvard.edu/privateurl.xhtml?token=9b6896f6-7ee2-4ce2-b4a5-f938e40de29f

S. rhizophila, 10% FlowClean cleaning agent: https://dataverse.harvard.edu/privateurl.xhtml?token=d0fc4221-f839-445b-b5ad-7be5c9aac958

S. rhizophila, 1% FlowClean cleaning agent: https://dataverse.harvard.edu/privateurl.xhtml?token=651518f9-a478-4933-b7a7-26b3dd53bb09

P. polymyxa, MilliQ H_2O: https://dataverse.harvard.edu/privateurl.xhtml?token=8b736fde-e942-4aca-a008-34b8291c45bf

P. polymyxa, 100% FlowClean cleaning agent: https://dataverse.harvard.edu/privateurl.xhtml?token=9d3dee5f-f7e8-4f24-8635-74ac06833e1e

P. polymyxa, 10% FlowClean cleaning agent: https://dataverse.harvard.edu/privateurl.xhtml?token=bffd285b-9828-4db8-8bd3-8b4b696cc474

P. polymyxa, 1% FlowClean cleaning agent: https://dataverse.harvard.edu/privateurl.xhtml?token=4717b913-3073-41c7-8035-8eec540061f8

Figure 4. Pre-fixed PFA/EtOH cells of the MC were analyzed online using the OC-300 coupled to the CytoFLEX. Three biological parallels were performed: https://dataverse.harvard.edu/privateurl.xhtml?token=b120f9de-02bd-4047-a308-22ff5b710736

SI 2.2. Comparison of mock community (MC) fingerprints after standard PFA/EtOH fixation and DAPI staining and after the new NaCl/NaN_3/EtOH fixation and DAPI staining method:
https://dataverse.harvard.edu/privateurl.xhtml?token=71bc6ba1-89ee-42f3-8761-0e7fd27ef98a

SI 2.4. Cell concentration over time of live cells of the MC:
https://dataverse.harvard.edu/privateurl.xhtml?token=4571de15-1b96-4247-9c91-8ec96f702219

SI 2.7 Comparison between DAPI patterns of the MC after PFA/EtOH fixation and NaCl/NaN3/EtOH fixation measured by the Influx:
https://dataverse.harvard.edu/privateurl.xhtml?token=9aece526-a485-4dff-b2e2-52026c44e479

References.

1. Cichocki, N., Hübschmann, T., Schattenberg, F., Kerckhof, F.-M., Overmann, J., & Müller, S. (2020). Bacterial mock communities as standards for reproducible cytometric microbiome analysis. *Nature Protocols*, *15*(9), 2788–2812. https://doi.org/10.1038/s41596-020-0362-0
2. Koch, C., Fetzer, I., Harms, H., & Müller, S. (2013). CHIC-an automated approach for the detection of dynamic variations in complex microbial communities. *Cytometry Part A*, *83A*(6), 561–567. https://doi.org/10.1002/cyto.a.22286
